# Forget For Now, but Remember Later: Can People Selectively Remove Information From Working Memory While Keeping it in Long-Term Memory?

**DOI:** 10.5334/joc.497

**Published:** 2026-04-07

**Authors:** Hannah Dames, Vencislav Popov, Klaus Oberauer

**Affiliations:** 1 University of Zurich, Switzerland

**Keywords:** Working memory, long-term memory, directed forgetting, intentional forgetting, removal

## Abstract

When instructed to remove a just-encoded item from working memory, people can do that very effectively. Here we investigate the side effects for episodic long-term memory of removing an item from working memory. Participants encoded lists of words into working memory, and each word was followed by a cue to remember or to forget that word. In a subsequent test of long-term memory, words to be maintained in working memory were remembered better than words to be removed. This was the case regardless of whether participants expected the long-term-memory test. In the final experiment we cued participants after each word to either remove it from working memory while keeping it in long-term memory, or to maintain it in working memory but forget it for the upcoming long-term memory test. Participants could not selectively remember information for one kind of memory test but not the other. Instead, they compromised, removing words less effectively from working memory than in the preceding experiments, and yet failing to remember those words better in the long-term memory test than the words they were told to forget for the long term. People cannot remove information from working memory and maintain it in long-term memory, or vice versa.

Can we intentionally forget information we no longer want to remember? Research on directed forgetting has addressed this question since the 1960s (Bjork, 1970; Bjork, LaBerge, & Legrand, 1968; Epstein, 1970), and found that, in many circumstances, we can decide to some extent, after studying some material, whether to remember or forget it. Whereas these pioneering studies investigated both short-term and long-term memory, subsequent work on directed forgetting has primarily focused on episodic memory. Directed forgetting has been studied mainly with two experimental paradigms, using list-wise and item-wise memory instructions. In the list-wise method, participants study a list of items (usually words) and are then instructed to forget that list. A control group, by contrast, is told to remember the list. Both groups then study a second list, after which their memory for both lists is tested (for a review see Sahakyan, Delaney, Foster, & Abushanab, 2013). In the item-wise method, participants study a single list of items and are instructed after each item whether to remember or to forget it. Memory is then tested for all items (Woodward & Bjork, 1971).

Evidence for the effectiveness of directed forgetting comes from two observations. First, memory for items that participants were initially instructed to forget is poorer than for items they were instructed to remember. Second, to-be-forgotten information interferes less than to-be-remembered information with other memory contents. For instance, when participants are asked to memorize a first list of words, and then told to forget it, their memory for a subsequently studied second list of words is better than when they have been told to remember the first list (Bjork, 1970). The facilitating effect of directed forgetting also extends to the item-wise method, where memory is superior for items that appear on a study list immediately after other to-be-forgotten items rather than after to-be-remembered items (Popov et al., 2019).

More recently, a second line of research has asked whether people can intentionally remove information from working memory (Lewis-Peacock, Kessler, & Oberauer, 2018; Oberauer, 2001). Removal has been studied with two methods, subset-wise removal and item-wise removal. In the subset-wise removal paradigm, participants initially encode two small sets of items into working memory (WM), after which they receive a cue indicating which subset to remember, implying that the other subset can be forgotten. A variable time after the cue, memory for the to-be-remembered set is tested. When the test follows the cue by 1 s or more, the set size of the to-be-forgotten subset no longer affects response times. If a subset is removed from WM, we should also expect memory accuracy to improve compared to a condition in which both subsets have to be remembered. This has been found in one study (Tortajada, Martínez-Pérez, Palmero, Fuentes, & Campoy, 2025) but not in another (Oberauer, 2018), casting some doubt on whether people can selectively remove a subset from WM after encoding both subsets.

The item-wise method for studying removal from WM is the same as the item-wise directed-forgetting method: After encoding each item, participants receive a cue whether to remember or forget that item. In tests of WM, item-wise removal is highly effective: When memory for the to-be-removed items is tested on a small subset of trials, it is very poor, though above chance (Dames & Oberauer, 2022). Moreover, when some items are cued as to be forgotten, memory performance for the remaining to-be-remembered items improves and becomes as good as in a control condition with the same number of to-be-remembered items but no additional to-be-removed items. This effect is independent of the time between a “forget” cue and presentation of the next item, and can therefore not be explained by decay of to-be-forgotten items (Oberauer, 2018).

The aim of the present work is to clarify the relation between control over the contents of WM and the formation of episodic memory traces for these contents. We use the item-wise method to induce the intention to remember or forget specific items because that method has been shown to effectively influence whether items are maintained in or removed from WM. We address two questions: (1) How does the maintenance or removal of information from WM affect its long-term retention? (2) Can people selectively remove information from WM while intentionally maintaining it in episodic LTM, and conversely, can they maintain information in WM while intentionally forgetting it from episodic LTM?

It would be advantageous if we could control whether or not to remember some information in WM and in episodic LTM independently. In daily life, we often need to briefly maintain and work with information that we know will never be relevant again later, for example, a provisional plan that we consider and then discard. Ideally, such information would not be remembered in the long term, as it might interfere with the plan we eventually adopt. In such a situation a person could maintain and manipulate the information in WM but not encode it into LTM (Popov & Dames, 2022). Conversely, we often receive information that we expect to be useful in the future but that is irrelevant – or even disruptive – to our current cognitive activity. For instance, while discussing an experimental design, a colleague might mention the location of that evening’s team dinner. In such situations, it would be beneficial to rapidly encode that information into episodic LTM while removing it from WM as quickly as possible.

That being said, there is reason to believe that intentionally maintaining or removing information in WM affects how well we can later retrieve it from episodic memory. Such a dependency could arise through at least two routes: First, information that the person intends to maintain is kept in WM longer than information they intend to remove. Longer maintenance in WM could lead to better encoding into episodic LTM. This could be either because the state of being held in WM itself is advantageous for the formation or strengthening of LTM traces, as assumed in the standard model of Atkinson and Shiffrin (1968). Maintenance in WM could also be advantageous for LTM because people might use free time to attend to items held in WM and elaborate them, which improves long-term retention (Craik & Tulving, 1975).

A second route through which a dependency in WM and LTM performance can occur is via shared representations and control processes. If WM and LTM rely on the same representations, as many theories of memory postulate (Cowan, 1995), then processes that change an item’s representation in WM are likely to impact episodic LTM as well. In our earlier work on item-based removal from WM (Dames & Oberauer, 2022) we found evidence for two processes: a rapid strengthening of item representations in response to a “remember” cue, and some process that weakens all items in WM. The net effect of these two processes is that to-be-remembered items are maintained whereas to-be-forgotten items are gradually removed from WM. It is plausible – though not necessary – that strengthening an item’s representation in WM also strengthens its representation in episodic memory as a side effect. For instance, to strengthen an item’s temporary bindings to its context in WM through Hebbian learning (Burgess & Hitch, 1999; Oberauer, Lewandowsky, Farrell, Jarrold, & Greaves, 2012), the item has to be activated together with its context. Jointly activating the item and its context also provides the prerequisite for associating them in episodic memory (for instance, through a similar, parallel Hebbian learning process).

Two further routes to a dependency between maintenance in WM and retention in LTM arise from the experimental set-up for investigating selective removal of information from WM. In the item-wise removal paradigm, an item followed by an R cue that instructs to remember the item (referred to as an “*R-item*” from here on) is likely to be attended until the next item is presented, whereas the person probably withdraws attention from an item followed by an F cue that tells them to forget that item (“*F-item*”). As a consequence, R-items receive initial attention more persistently. We refer to the duration of initial attention as the duration for which an item is attended right after its presentation, to distinguish it from the time for which attention might be later re-directed to an item after encoding further items, for instance to elaborate it (as discussed above). As the duration of attention is a determinant of the formation of episodic memory traces (Craik, Govoni, Naveh-Benjamin, & Anderson, 1996), longer initial attention should translate into better episodic memory for R-items. This confound between the memory cue (R vs. F) and the duration of initial attention can be controlled by varying the interval between the item and the subsequent cue. Finally, in experiments on selective removal of information from WM there is an immediate memory test for the R-items but not for the F-items. Thereby, episodic LTM for to-be-maintained items is likely to benefit from the retrieval practice in the immediate memory test (Sutterer & Awh, 2016). This confound can be controlled by including only part of the R-items in the immediate test of WM and use the remaining items in the subsequent test of episodic LTM. In the experiments reported here we address both these confounds, so that any dependency between directed forgetting in WM and the retention in episodic LTM can be attributed to the first two routes, the duration of maintenance in WM, or side effects of the processes for maintaining and removing in WM for episodic-memory traces.

Experiments 1 and 2 served to investigate whether removing information from WM makes it less accessible in a later test of LTM. In these experiments LTM for the stimuli of the WM test was formed incidentally as participants did not expect a delayed test. With Experiments 3 to 5 we ask whether people can intentionally remember information in the long term while removing it from WM when the delayed test is announced upfront. In Experiment 6 we instructed participants directly to remove information from WM while maintaining it in LTM, or conversely, maintain information in WM while forgetting it over the long term.

## Experiment 1

The goal of Experiment 1 was to investigate the long-term effects of directed forgetting in WM. We ask whether information that is maintained in WM is also remembered better in the long-term than information that was forgotten for the WM test. To this end, we added a surprise LTM test at the end of a series of trials of the WM task.

**Figure 1:**
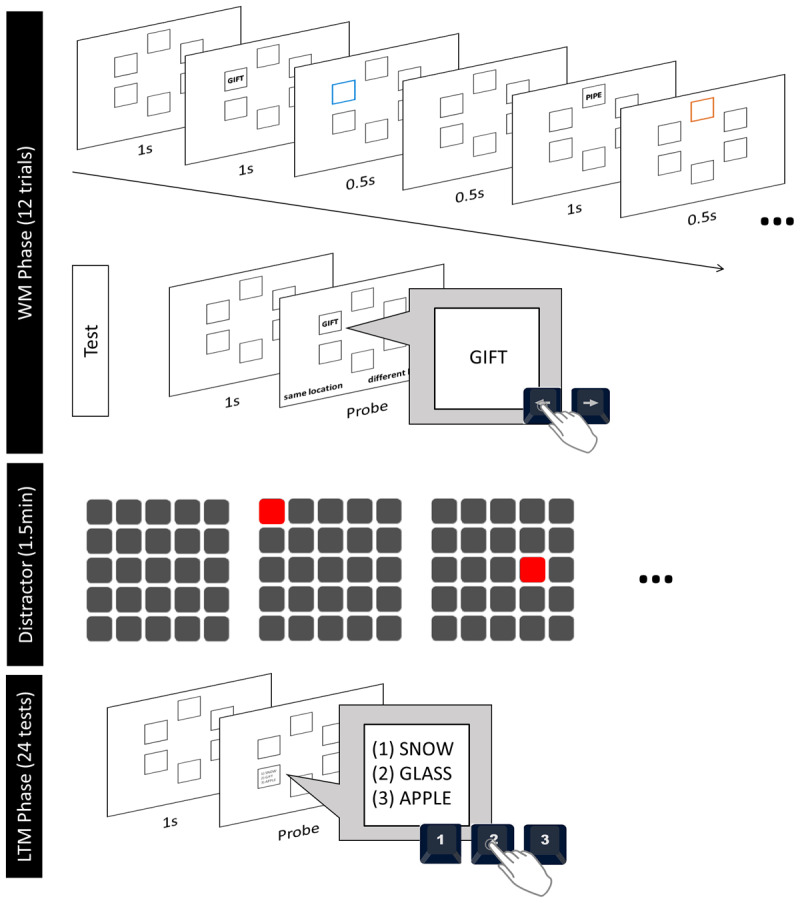
Procedure and General Trial Structure.

We used the item-wise directed forgetting WM task of Dames and Oberauer (2022) with a few modifications. The procedure is illustrated in Figure 1. In each trial of the WM task participants are asked to remember seven words for an immediate test. The words are presented in clockwise order in a circular array of frames. Presentation of each word is followed by an R or an F cue. A random subset of the R-words is tested immediately after presentation. Different from Dames and Oberauer (2022), we never tested the F-items in the immediate test, because here our interest was primarily on long-term memory for these words. After 12 trials of the WM task, and a distractor period, we tested episodic LTM for the to-be-remembered and to-be-forgotten words from the WM phase.

The R or F cues are presented for 0.5 s, followed by a 0.5 s blank interval before the onset of the next word. Assuming that to-be-remembered words are attended persistently until the next word appears, whereas attention is immediately withdrawn from to-be-forgotten words, a to-be-remembered word is attended up to 1 s longer than a to-be-forgotten word. To control for this confound between cue and attention duration, we varied the stimulus-cue interval (SCI) between each word and the following R/F cue (0 vs 1 s between word offset and cue onset). In this way, F-items after a long SCI are attended to at least as long as R-items after a short SCI.

### Method

Methods, hypotheses, and the data analysis procedures were preregistered; the preregistration document can be found online (https://osf.io/n3w2q).

#### Participants and Exclusion Criteria

150 native English speakers completed the whole experiment via the web-based participation platform Prolific. After applying the following preregistered exclusion criteria, the final sample consisted of 134 participants (*M*_age_ = 26.0, *SD*_age_ = 4.6, range 18–34 years; 57.9% female, 40.6% male): We excluded participants who indicated that they did not follow the instructions or could not correctly state what they were supposed to do when the frames turned blue/orange (*n* = 1). Furthermore, we excluded participants who stated that they did not participate seriously in the experiment or did not agree that we could use their data (*n* = 2), with a mean error rate higher than 30% in the WM test phase (*n* = 11), who indicated that they did not try to respond correctly when they noticed they were being tested for to-be-forgotten words in the LTM test (*n* = 1), or who had technical issues during completion (*n* = 1).

All participants were naive to the purpose of the experiment and had normal or corrected-to-normal vision. Participants signed an informed consent form prior to the study, and they were debriefed at the end. The study protocol is in line with the guidelines of the institutional ethics review board.

#### Material

The stimuli were frequent English nouns with a length of 4–5 letters. For every participant 396 words were randomly drawn from a pool of 600 words. For each trial of the WM phase we drew 7 words at random to form the memory set. No word was used more than once throughout the WM phase. Additional words from the pool were used as not-presented lures in the LTM test. The experiment was programmed using *jsPsych* (de Leeuw, 2015).

#### Procedure

We used the item-wise directed-forgetting procedure for the WM test, which produced robust directed forgetting effects in WM (Dames & Oberauer, 2022), combined with a subsequent LTM test. There were three phases: A WM phase, a distractor phase, and a LTM test phase.

##### Working memory phase

The trial structure of the WM phase is illustrated in Figure 1: First, participants were presented with seven squared frames in thin black outlines on a white background. The frames were arranged 360/7° apart from each other on a circle. Following the presentation of all seven empty frames for 1s, seven words were displayed one by one for 1s each in a clockwise order. The first word was presented on the top left at 10 o’clock. After each word offset, all frames remained empty for either 0s or 1s depending on the current SCI condition. After the SCI, the frame of the just-presented word turned either blue or orange for 0.5s, indicating a to-be-remembered or to-be-forgotten word, respectively. After the offset of the memory cue the frames remained empty for 0.5s before the subsequent word was presented in the next frame. Participants were instructed to remember only the words in a blue frame for the immediate memory test. They were told to try to forget the words in an orange frame, as this would make it easier for them to remember the words in the blue frames.

Following the display of the last word and memory cue, a retention interval of 1s followed. The trial ended with a local-recognition probe where one randomly selected to-be-remembered word of that trial was presented in one of the to-be-remembered frames. That is, we never tested participants’ memory for one of the to-be-forgotten words throughout the WM phase. Using the left and right arrow keys, participants indicated whether a presented word was the same as the one originally presented in that frame (match, 50%) or not (mismatch, 50%). A match probe was the word from that frame; a mismatch probe was a word from another to-be-remembered frame in that trial.

There were twelve trials in the WM phase. To achieve 50% to-be-forgotten items on average, we distributed trials evenly among two set-size conditions: set-size 7–3 and set-size 7–4. In the set-size 7–3 condition, three randomly chosen words were followed by a blue (R) frame, and the other four words by an orange (F) frame. In the set-size 7–4 condition, four randomly chosen words were followed by a blue (R) frame, and the other three words by an orange (F) frame. Within each set-size condition, three trials were randomly allocated to each SCI condition (0s vs. 1s). Within each set-size + SCI combination, match and mismatch probes were assigned at random among the three trials with the constraint that there were no more than two match, or two mismatch trials, and there were equally many match and mismatch trials across all 6 trials of each memory-cue (R vs. F) condition.

Participants received instructions about the WM task at the beginning of the experiment. They were not informed about the later LTM test at this point. They were instructed not to use any external help (e.g., use pen and paper) and to respond as quickly and accurately as possible. Following the instructions, participants practiced the WM task (six trials in total: three trials with each SCI level; for each SCI condition there were two trials for set-size 7–3 and two trials for set-size 7–4, all presented in random order). In the practice block, participants received feedback (“correct”/“incorrect”) after each response, but they received no performance feedback for the remainder of the experiment. Following the practice block, participants had the opportunity to reread the instructions and redo the practice block if needed or proceed with the experiment. Next, the test trials of the WM phase followed.

##### Distractor phase

After the twelve WM trials, in a distractor phase intended to purge WM, participants solved a visual working memory task for 90 seconds. In each trial a sequence of *n* squares in a 5 × 5 matrix lit up, each for 1 s. Participants’ task was to click on the squares in the same order as they had lit up. The task started with a set size of *n* = 3, which was adapted: After each correctly reproduced sequence, *n* was increased by one; after each sequence with an error, *n* was decreased by one.

##### LTM phase

At the beginning of the LTM phase, we informed participants that we would now also test their memory for to-be-forgotten words. In such cases, participants were instructed to do their best to remember the to-be-forgotten words. We tested participants’ memory for one to-be-remembered and one to-be-forgotten word from each of the WM trials using a three-alternative forced-choice (3-AFC) test. The 24 trials were presented in random order. We only tested words that had not been tested during the WM phase to avoid a confound with retrieval practice. If the WM test had been a mismatch test, we excluded the word shown as probe in the WM test. For the 3 AFC test we presented the seven frames of the WM task again, and in one of the frames three test words were presented: (1) the target word that had been originally presented in the frame (“old-match”), (2) another word that had been presented in a different frame in the same WM trial (“old-mismatch”), and (3) a completely new word (“new”). When the old-match word was a to-be-remembered word of the WM phase, the old-mismatch word was from another to-be-remembered frame; when the old-match word was a to-be-forgotten word, the old-mismatch word likewise came from another to-be-forgotten frame. Using the keys [1], [2], and [3] participants were instructed to select the word that was the same as the one originally presented in the frame (“old-match”).

The 3-AFC test provided information about item memory (i.e., memory for which items have been presented) and binding memory (i.e., memory for the relation between an item and its frame in the WM test), enabling us to assess directed-forgetting effects on both aspects of memory separately. Participants’ probability to select an old over a new word [p(old)] in the LTM test served as a measure for item memory. Binding memory was measured by participants’ probability to select the match word, given they selected an old word [p(match|old)]. If directed forgetting weakened item memory, participants should struggle to discriminate between new words and the two old but to-be-forgotten words. If directed forgetting impaired memory for item-context bindings, participants should struggle to correctly discriminate between the old match and the old mismatch word.

Using post-experimental questions, we checked to what extent participants followed the task instructions (e.g., what they did when they saw an orange or blue frame) and asked them to indicate and describe how they responded when to-be-forgotten words were tested during the LTM phase. Furthermore, we asked them whether they used external help to remember the words, and whether they participated seriously in the experiment.

#### Data Analysis

We analyzed the correctness of participants’ responses in the LTM test as a function of memory cue (R vs. F) and SCI through Bayesian generalized linear mixed models (BGLMMs) using the *R* package *brms* (Bürkner, 2025). To evaluate the correctness of item memory we coded the choice of old-match or old-mismatch words as correct (1) and choices of the new word as error (0). To evaluate correctness of binding memory we coded the choice of old-match words as correct (1), the choice of old-mismatch words as error (0), and excluded trials in which new words were chosen by setting the response to missing. We assumed a Bernoulli data distribution predicted by a linear model through a logistic link function.

All our models included random intercepts for participants as well as random slopes for all within-subjects effects (except the highest-order interaction). For the regression coefficients we used moderately informative Cauchy priors centered on 0 with a scale of 0.353 (i.e., default priors proposed for hypothesis testing with logistic models; Oberauer, 2019). For random effects, we used non-negative weakly informative priors (half student t-prior with three degrees of freedom and a scaling parameter of 2.5). All categorical predictors were coded as sum-to-zero contrasts, and continuous predictors were mean-centered.

We estimated the posteriors by sampling parameter values using the No-U-Turn Sampler (NUTS, an extension of the Hamilton Monte Carlo sampling method) as implemented in Stan (Carpenter et al., 2017). We sampled through eight independent Markov chains with 30,000 iterations each (1,000 warm up each). To investigate convergence, we inspected Rhat values (ratio of between-chain variance to within-chain variance). Rhat values were ≤ 1.01 for all parameters in every model. We estimated Bayes Factors (BFs) through the Savage-Dickey approximation (Wagenmakers, Lodewyckx, Kuriyal, & Grasman, 2010). This involved fitting the full model including all fixed and random effects. For each effect of interest we computed the Savage-Dickey density ratio as the ratio between the densities of the prior to the density of the posterior of the effect evaluated at zero. The density of the posterior was the density of the normal distribution with mean and standard deviation set to the mean and the standard deviation of the MCMC samples. This ratio is an estimate of the Bayes factor in favor of the tested effect and against the null hypothesis for that effect, which we denote BF_10_. The inverse ratio estimates the Bayes factor in favor of the null hypothesis, which we denote BF_01_. Generally, a BF larger than 3 can be considered as substantial evidence for one hypothesis over the other. For instance, a BF_10_ of 3 would indicate that the data are 3 times more likely under the alternative hypothesis than under the null hypothesis.

To test whether the memory for to-be-forgotten words was better than chance, we compared two “intercept-only”-BGLMMs on the correctness of participants’ responses on these trials. One model included a free parameter for the intercept. For the other model the intercept parameter was fixed at chance-level performance (0.67 for item memory, and 0.50 for binding memory). We tested whether the model including a free intercept was superior to the model where the intercept was fixed.

### Results

**Figure 2:**
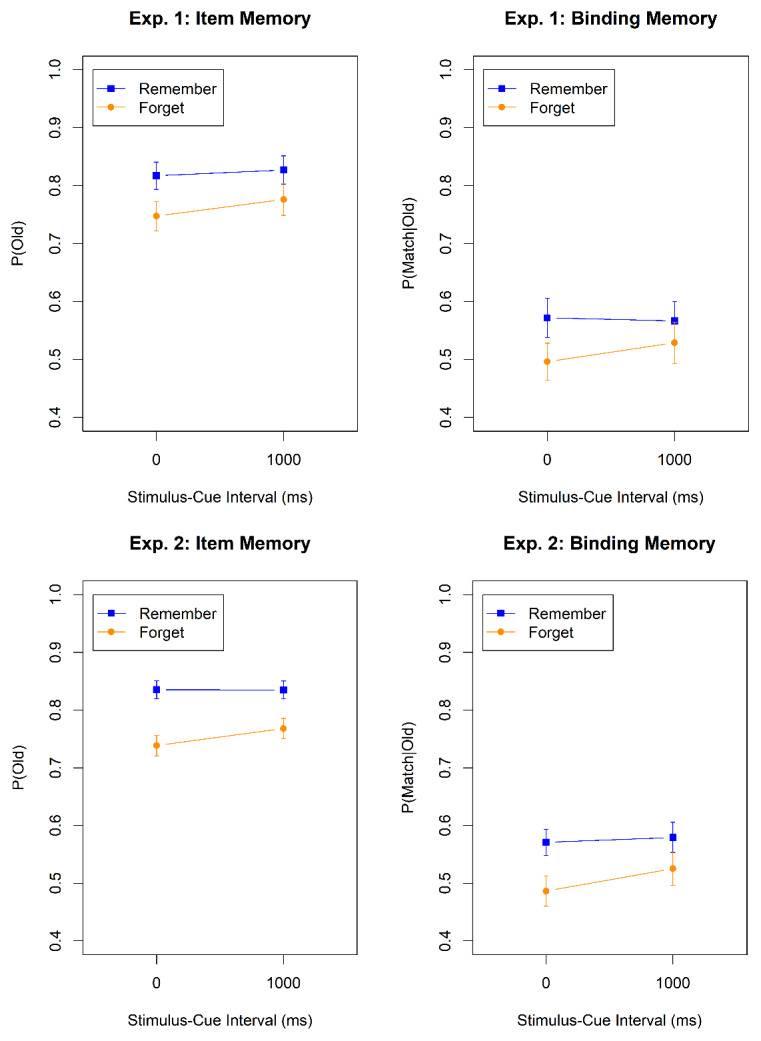
Item Memory and Binding Memory in the Long-Term Memory Tests of Experiment 1 and Experiment 2. *Note*. Error bars represent the within subject 95% confidence intervals. Remember = words cued to be remembered in the WM test; Forget = words cued to be forgotten in the WM test.

Proportion correct on the WM test was high (M = 0.96, SD = 0.07) with no discernable differences between the SCI conditions. Mean correctness scores of item and binding memory in the LTM test are displayed in Figure 2 (top). Table 1 summarizes the Bayes factors for the hypothesis tests. There was evidence for a main effect of memory instruction (i.e., whether to remember or to forget a word) on both item and binding memory. P(old) was consistently lower for to-be-forgotten than for to-be-remembered words, indicating long-term effects of directed forgetting in WM for item-memory. Still, item memory for to-be-forgotten words was above chance level (76.2%; BF_10_ = 1.2 × 10^11^). We also observed long-term directed forgetting effects in binding memory: p(match|old) was lower for to-be-forgotten than for to-be-remembered words. Binding memory for to-be-forgotten words was indistinguishable from chance level (51.2%; BF_10_ = 0.059). There was evidence against a main effect of SCI, and against an interaction of the memory instruction with SCI.

**Table 1:** Bayes Factors for Experimental Effects in Experiments 1 and 2.


DEPENDENT VARIABLE	MEMORY INSTRUCTION	SCI	INTERACTION

Exp. 1: Item Memory	5.6	0.10	0.14

Exp. 1: Binding Memory	282	0.21	0.11

Exp. 2: Item Memory	1.7 × 10^8^	0.12	0.96

Exp. 2: Binding Memory	39	0.09	0.09


To summarize, in Experiment 1, we observed long-term effects of directed forgetting in WM for both item and binding memory. The time for which a word had to be held in WM before the memory cue was given had no effect on LTM. This renders it unlikely that the directed-forgetting effect on LTM came about because a forget cue curtailed the time for which the item was attended to or held in WM. These long-term effects were a byproduct of WM instructions. Unlike the traditional item-wise LTM directed forgetting procedure, our participants had no reason to encode any of the words, regardless of their cue, into LTM.

## Experiment 2

Experiment 1 already controlled for retrieval practice on LTM by probing only those items in the LTM phase that had not been tested in the WM task. However, it is possible that retrieval practice generalizes to all items in WM: Testing any one of the to-be-remembered words in the WM phase could have boosted memory for all the to-be-remembered words in that trial, including not-tested words. Potentially this resulted in better long-term memory for all to-be-remembered words, resulting in the observed directed forgetting effect in LTM. The aim of Experiment 2 was to confirm or rule out this alternative explanation. Only 50% of the trials in the WM phase were followed by a test. We then examined the directed forgetting effects for words that were presented in WM trials without a WM test.

### Method

Methods, hypotheses, and the data analysis procedures were preregistered and can be found online (https://osf.io/z7kes).

#### Participants

The same preregistered inclusion and exclusion criteria as in Experiment 1 were applied and we only recruited participants that had not participated in the previous experiment. From the initial sample (*N* = 294), 8 participants had to be excluded, resulting in a final sample size of *n* = 286 participants (*M*_age_ = 26.8, *SD* = 4.9, range: 18–35 years; 60.5% female, 38.1% male, 1.4% diverse).

#### Material and Procedure

Stimuli and procedure were identical to Experiment 1 with the exceptions that only half of the trials in the WM phase included a memory test.

### Results

Performance in the WM test was again high (M = 0.91, SD = 0.14). Mean accuracy rates in the LTM test can be taken from Figure 2 (bottom). The statistical analysis procedures were identical to Experiment 1 but we only analyzed LTM test performance for words that belonged to a WM trial not followed by an immediate test.

Item memory was lower for to-be-forgotten than for to-be-remembered words, indicating long-term effects of directed forgetting in WM. Again, item memory for to-be-forgotten words was above chance level (75.1%; BF_10_ = 2.2 × 10^8^). We also observed long-term directed forgetting effects for binding memory: P(match|old) was lower for to-be-forgotten than for to-be-remembered words. Binding memory for to-be-forgotten words was at chance level (50.2%; BF_10_ = 0.036). There was again evidence against a main effect of SCI; the evidence for the interaction was ambiguous for item memory, but clearly against the interaction for binding memory. In summary, we replicated the long-term effects of directed forgetting in WM even for trials without a memory test in the WM phase.

## Directed-Forgetting Effect Controlling for Initial Attention Duration

On the very conservative assumption that people withdraw attention from a word instantly upon the onset of a forget cue and continue attending to it until the onset of the next word when seeing a remember cue, to-be-remembered words are attended to for 1 s longer than to-be-forgotten words. To control for that difference in the duration of initial attention, we compared LTM for to-be-remembered words followed by a short SCI and to-be-forgotten words followed by a long SCI. As this reduces the number of trials in each condition, we pooled the data from Experiments 1 and 2 and ran a logistic mixed-effects model with memory instruction (remember vs. forget) and experiment as predictors. This analysis confirmed that to-be-remembered words fared better than to-be-forgotten words in the LTM test with respect to item memory (M = 0.84, SD = 0.21 vs. M = 0.77, SD = 0.24; BF_10_ = 9.07) and binding memory (M = 0.58, SD = 0.29 vs. M = 0.52, SD = 0.32; BF_10_ = 3.22).

## Experiment 3

In Experiments 1 and 2, we demonstrated long-term effects of directed forgetting in WM. That is, items cued to be removed from WM are remembered worse both in an immediate test of WM (see Dames & Oberauer, 2022) as well as in delayed test of episodic LTM. In the first two experiments episodic LTM was incidental, because participants did not expect the delayed test.

Can people intentionally maintain information in episodic LTM while removing it from WM? If that is the case, the effect of directed forgetting in WM on subsequent LTM should be substantially mitigated when people expect the LTM test than when they don’t. At the same time, removal from WM should still be equally effective in both expectation conditions. By contrast, if people cannot remove information from WM without side effects on LTM, then we should expect one of two scenarios:^1^ When participants prioritize the instruction to remove some items from WM, we should find equally strong directed-forgetting effects for expected and unexpected LTM tests. When participants prioritize maintenance in LTM when they expect an LTM test, then we should find that the instruction to remove some items from WM is less effective when they expect an LTM test than when they don’t.

In Experiments 3 and 4 we manipulated participants’ expectation for a LTM test: For half of the participants the LTM test at the end of the experiment came as a surprise, just like in Experiments 1 and 2. These participants should not be motivated to remember the TBF information – neither in the short-term nor in the long-term. The other half of the participants were told that there will be a LTM test for all words, regardless of the R or F cues for the immediate test. These participants were instructed to forget the words followed by an F cue for the WM test but not the LTM test. We investigated whether the directed-forgetting effect in WM was reduced for participants in the test-expectation group compared to the no-test-expectation group. Through an LTM test at the end of the experiment we tested whether expectation of this test influenced to what degree the directed-forgetting manipulation in WM affected episodic LTM.

### Method

Methods, hypotheses, and the data analysis procedures were preregistered and can be found online (https://osf.io/drh98).

#### Participants

The same preregistered inclusion and exclusion criteria as in the preceding experiments were applied, and we only recruited participants that had not participated in the previous experiments. From the initial sample (*N* = 146), 11 participants had to be excluded, resulting in a final sample size of *n* = 135 participants (*M*_age_ = 27.6, *SD*_age_ = 4.8, range 19–35 years; 48.9% female, 41.1% male), with 73 participants that expected the LTM test and 62 participants that did not.

#### Material and Procedure

There were three phases: A WM test phase, a distractor phase, and a LTM test phase. Different from Experiments 1 and 2, we were now interested in participant’s performance in the WM task. Thus, we included more (39) WM trials.

The instructions for the WM phase stressed the importance of remembering only the words followed by an R cue for the immediate memory test, and that participants should try to forget the words followed by an F cue. We informed them that on very few occasions their memory for to-be-forgotten words would be tested. In such cases, participants were instructed to do their best to remember the to-be-forgotten words. They were told that they should nevertheless try their best to forget all items in an orange frame, as this would make it easier for them to remember the items in the blue frames and therefore put them at an advantage. Participants were instructed not to use any external help (e.g., use pen and paper) and to respond as quickly and accurately as possible.

##### Working memory test

The procedure for the WM test was the same as in the preceding experiments, with the exception of the test. WM was tested with a 2-step procedure. The first step was a 2-AFC test in which the tested word from the memory set and one completely new word were presented side by side in the center of the screen. Using their computer mouse, participants selected the word that was presented in that trial. For the second step, the not-selected word disappeared, and participants were instructed to click on the frame that the selected word had been presented in. This test yields separate estimates for item and binding memory. We measured item memory as the probability of selecting the target word in the first test, p(Old), and binding memory as the probability of selecting the correct frame, if the target was chosen in the first step, p(Match|Old).

There were four conditions. In the *large set-size* condition, participants were instructed to remember all seven words. On those trials, every word was followed by a R cue (blue frame), and memory for one randomly selected word was tested. For the *small set-size* condition, 2, 3, or 4 randomly chosen words were replaced by “XXXX”, each displayed in the same way as the words. The “XXXX”-stimuli were always followed by an orange (F) frame. The other words were followed by a blue (R) frame. Memory for one randomly selected word was tested. In the *removal* condition, 3, 4, or 5 randomly chosen words were followed by a blue (R) frame, and the remaining words by an orange (F) frame. For trials in the *removal remember* condition, memory for one randomly selected to-be-remembered word was tested. For *removal forget* condition trials, memory for one randomly selected to-be-forgotten word was tested. There were 12 small set-size trials, 12 large set-size trials, 12 removal trials (R-item tested in WM) and 3 removal trials (F-item tested in WM).

Following the instructions, participants had the chance to practice the WM task. Practice comprised 2 small set-size trials, 2 large set-size trials, and 2 set-size removal trials with tests of a to-be-remembered word, presented in random order. In the practice block participants received feedback (“correct”/“incorrect”). They received no feedback on their performance for the remainder of the experiment. After the practice block, participants had the option to reread the instructions and redo the practice block or proceed with the experiment. Next, the 39 test trials of the WM task followed.

##### LTM test

At the beginning of the WM phase, we manipulated participants’ expectations for a LTM test. For half of the participants the LTM test was not mentioned at this point. The other half of participants were informed about the LTM test, announcing that it would test both words followed by R and by F cues in the WM phase. We told participants that they should nevertheless try to forget all items in an orange frame for the WM test while still trying to remember them for the LTM test.

After the WM phase, in a distractor phase intended to purge WM, participants solved a visual working memory task for 1.5 minutes. Next, we tested memory for one to-be-remembered and one to-be-forgotten word from each WM trial (thus, 78 tests in total), using the same 2-step test procedure as in the WM phase. We introduced this change in the LTM test relative to the first two experiments to make the LTM test more comparable to the WM test. At the beginning of the LTM test phase, we informed participants that now also their memory for to-be-forgotten words would be tested. In such cases, participants were instructed to do their best to remember the words.

Using post-experimental questions, we checked whether participants had any suspicion about the purpose of the experiment or the directed-forgetting instruction. We took special care to additionally check whether participants correctly understood the task by asking them about their comprehension of the instructions and the task. Specifically, we asked what they were supposed to do and what they actually did when they saw the blue frame, and the orange frame. Participants stated if and (if so) how often they noticed to-be-forgotten words being tested. They also described what they thought they were supposed to do when to-be-forgotten words were tested and whether they tried to respond correctly on those trials. We additionally assessed how they responded on tests of to-be-forgotten words using a multiple-choice question. Furthermore, we assessed how strongly participants tried to remember/forget the words on Likert scales. Last, we asked them whether they used external help to remember the words. Participants were accurately debriefed about the reason behind testing both to-be-remembered and to-be-forgotten items.

### Results and Discussion

**Figure 3:**
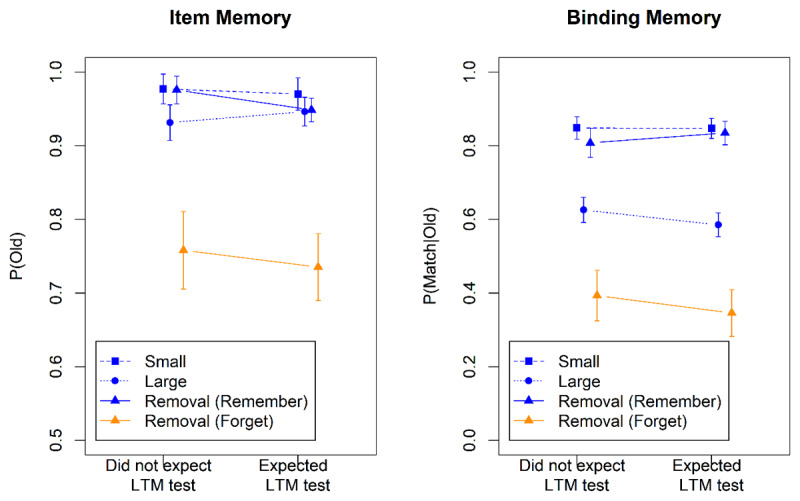
Accuracy in the WM Test of Experiment 3.

Figure 3 shows accuracies from the WM test. We can gauge the effectiveness of removal of the to-be-forgotten words from these data in two ways. First, effective removal should render memory for to-be-forgotten words much poorer than for to-be-remembered words (Dames & Oberauer, 2022). Second, memory for the to-be-remembered words from the removal condition should be better than in the large set-size condition. To the extent that removal is effective, it should approximate the performance level of the small set size (Oberauer, 2018).

To test these predictions, we defined three contrasts described in Table 2; each contrast serves to test one prediction through the relevant pairwise comparison of two conditions. Because the Removal-Small and the Removal-Large contrasts are not orthogonal, we tested them in separate analyses. Therefore, we ran one logistic mixed-effects model with Removal-Small, Directed Forgetting, and Group as predictors, and a second model in which Removal-Large replaced Removal-Small.

**Table 2:** Contrasts for Analysis of Experiments 3 and 4.


CONTRAST	SMALL SET SIZE	LARGE SET SIZE	REMOVAL (TBR)	REMOVAL (TBF)

Removal-Small	1	0	–1	0

Removal-Large	0	1	–1	0

Directed Forgetting	0	0	1	–1


Table 3 summarizes the Bayes factors for the two analyses.^2^ WM for the to-be-remembered items in the removal condition was much better than for the large set-size condition, but also somewhat worse than the small set-size condition, showing that removal was highly but not completely effective. The interaction of the Removal-Large contrast with Group suggests that, with respect to item memory, removal was more effective when participants did not expect the LTM test; no such interaction was found with respect to binding memory. Memory for the to-be-forgotten items in the removal condition was much worse than in all other conditions, as reflected in the main effect of the Directed-Forgetting contrast, confirming that removal was effective. There was no evidence that the Directed-Forgetting contrast interacted with Group.

**Table 3:** Bayes Factors for Working Memory and Long-Term Memory Performance in Experiment 3.


EFFECT	WORKING MEMORY		LONG-TERM MEMORY	
	
	ITEM MEMORY	BINDING MEMORY	ITEM MEMORY	BINDING MEMORY

Removal-Small	1.8·10^13^	2.3·10^45^	0.07	0.77

Removal-Large	9.5	4.3·10^7^	0.08	0.48

Directed Forgetting	3.1·10^31^	1.1·10^11^	0.77	10.4

Group	0.36	0.26	0.08	0.20

Removal-Small × Group	0.38	0.23	0.20	0.15

Removal-Large × Group	22	0.23	0.07	0.11

Directed Forgetting × Group	0.29	0.6	0.08	0.46


**Figure 4:**
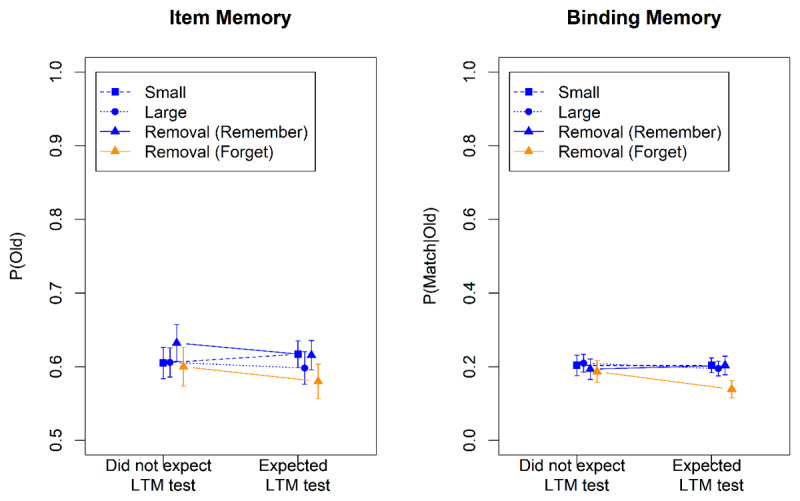
Accuracy in the Long-Term-Memory Test of Experiment 3.

Performance in the LTM test is shown in Figure 4. For the analysis we used the same contrasts and the same pair of logistic models as for the WM test. The results are summarized in Table 3. There was evidence for a directed-forgetting effect on binding memory: Items to be removed from WM were remembered less in the LTM test than items to be maintained. Figure 4 suggests that this effect was more pronounced when participants expected the LTM test, but the interaction of the Directed-Forgetting effect with Group was not supported statistically. No directed-forgetting effect was observed for item memory. The set-size in the WM test had no impact on the probability of recalling a word in the LTM test, in line with previous findings (Bartsch, Loaiza, & Oberauer, 2019).

We conclude that participants were highly effective in removing just-encoded items from WM, replicating Dames and Oberauer (2022). Those who expected an LTM test might have been less effective in removing item information from WM than participants not expecting such a test, though evidence for that conjecture comes from only one of the three indicators of effective removal (i.e., the Removal-Large contrast). We found no effect of test expectancy on the effectiveness of removal of binding information. As in the preceding experiments, removing information from WM reduced the chance of remembering it in the long term. This directed-forgetting effect was not measurably influenced by test expectancy.

However, in the post-experimental questionnaire, most participants reported feeling overwhelmed by the number of words, causing them to focus primarily on the immediate memory test. They indicated that they did not use any specific strategies to remember the words for the LTM test, resulting in long-term retention being largely neglected in favor of immediate task performance. We address that limitation in Experiment 4.

## Experiment 4

We adjusted the procedure of Experiment 3 to encourage participants to try more seriously to remember items for the LTM test. Specifically, we limited the experiment to nine WM trials (six small set-size and six removal trials) so that the number of words participants had to remember for the LTM test was substantially reduced compared to Experiment 3. Moreover, we never tested to-be-forgotten items in the immediate test. We assessed the effectiveness of removal from WM by testing whether performance in the removal condition approximates that in the small set-size condition.

### Method

Methods, hypotheses, and the data analysis procedures were preregistered and can be found online (https://osf.io/pt7ys).

#### Participants

The same preregistered inclusion and exclusion criteria as in the preceding experiments were applied, and we only recruited participants that had not participated in the previous experiments. From the initial sample (*N* = 312), 29 participants had to be excluded, resulting in a final sample size of *n* = 283 participants (*M*_age_ = 28.1, *SD*_age_ = 4.6, range 18–38 years; 50.9% female, 45.9% male, 3.2% diverse), with 147 participants that expected the LTM test and 136 participants that did not.

#### Material and Procedure

Stimuli and procedure were identical to Experiment 3 with the following exceptions: First, following the instructions, participants had the chance to practice the WM task for three trials. Second, we only included nine WM trials (three small set-size and six removal trials), and we never tested a to-be-forgotten item. In the LTM phase, we tested memory for one to-be-remembered and one to-be-forgotten word from each WM trial (thus, 18 tests in total), using the same two-step test procedure as in the WM phase.

### Results and Discussion

**Figure 5:**
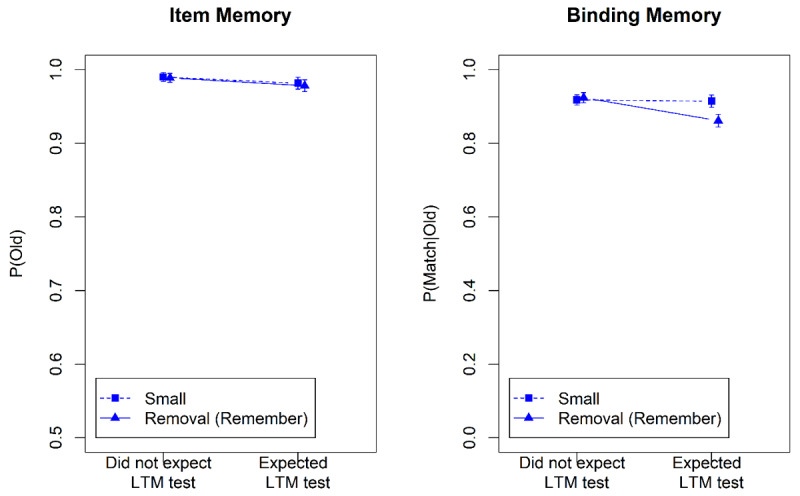
Accuracy in the Working-Memory Test of Experiment 4.

**Figure 6:**
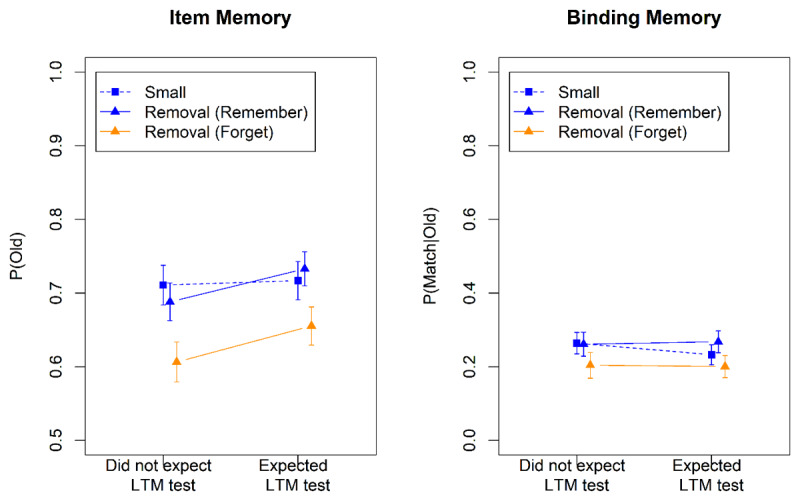
Accuracy in the Long-Term-Memory Test of Experiment 4.

Figure 5 shows the results from the WM tests, and Figure 6 the results of the LTM test. We analyzed the data using the subset of contrasts from Table 2 that could be computed in this experiment. The Bayes factors are summarized in Table 4. Once again there was no evidence for a main effect of group, or for any interaction involving group, though one Bayes factor reflected weak evidence in favor of an interaction of group with the Remember-Small contrast in the WM test. This weak interaction hints at less effective removal in the group that expected an LTM test.

**Table 4:** Bayes Factors for Working Memory and Long-Term Memory Performance in Experiment 4.


EFFECT	WORKING MEMORY		LONG-TERM MEMORY	
	
	ITEM MEMORY	BINDING MEMORY	ITEM MEMORY	BINDING MEMORY

Removal-Small	0.38	0.66	6.9	0.77

Directed Forgetting			3.1·10^6^	83.5

Group	0.53	0.17	0.53	0.17

Removal-Small × Group	0.35	2.21	0.25	0.18

Directed Forgetting × Group			0.11	0.15


In both groups, words that were removed from WM were recalled worse in the LTM test, as reflected in strong evidence for the Directed-Forgetting contrast. This effect did not differ between groups. Participants who expected the LTM test were not willing, or not able, to protect LTM from the side effects of removing the to-be-forgotten items from WM.

The finding that expecting a delayed memory test does not improve LTM is in agreement with previous findings (Labaronne, Ferreri, & Plancher, 2024). More generally, it is related to the long-standing finding that when the learning material is processed “deeply” (i.e., semantically), whether people intend to remember it or not has no effect on episodic memory (Hyde & Jenkins, 1969; Oberauer & Greve, 2021), though this holds only for list-wise or group-wise manipulations of intention (Popov & Dames, 2022). The new aspect of this null effect in the present experiments is that it holds also for F items, for which it is not obvious that they have been processed deeply.

## Experiment 5

In Experiments 3 and 4, we found that expectations about a LTM test did not affect long-term directed forgetting effects. One possible explanation is that, even if participants are capable of independently controlling maintenance in WM and LTM, they may not have had sufficient time to encode a to-be-forgotten item into episodic LTM before it was removed from WM. With more time to form an LTM trace before receiving the cue to remove (or maintain) an item, it might be possible to remove information from WM without impairing one’s ability to remember it in the long term. To test this possibility, in Experiment 5 we tested whether LTM test expectation affects the magnitude of long-term directed-forgetting effects when providing participants with much more time before the memory cue was presented.

### Method

Methods, hypotheses, and the data analysis procedures were preregistered and can be found online (https://osf.io/6qztx).

#### Participants

The same preregistered inclusion and exclusion criteria as in the preceding experiments were applied, and we only recruited participants that had not participated in the previous experiments. From the initial sample (*N* = 317), 48 participants had to be excluded (note, that 40 participants had to be excluded, because they had a mean error rate higher than 20%), resulting in a final sample size of *n* = 269 participants (*M*_age_ = 27.8, *SD*_age_ = 4.6, range 18–36 years; 46.8% female, 50.6% male, 2.2% other, 0.4% preferred not to say), with 137 participants that expected the LTM test and 132 participants that did not.

#### Material and Procedure

Stimuli and procedure were identical to Experiment 4 but this time we provided 5 s between word off-set and the onset of the memory cue (R vs. F).

### Results

**Figure 7:**
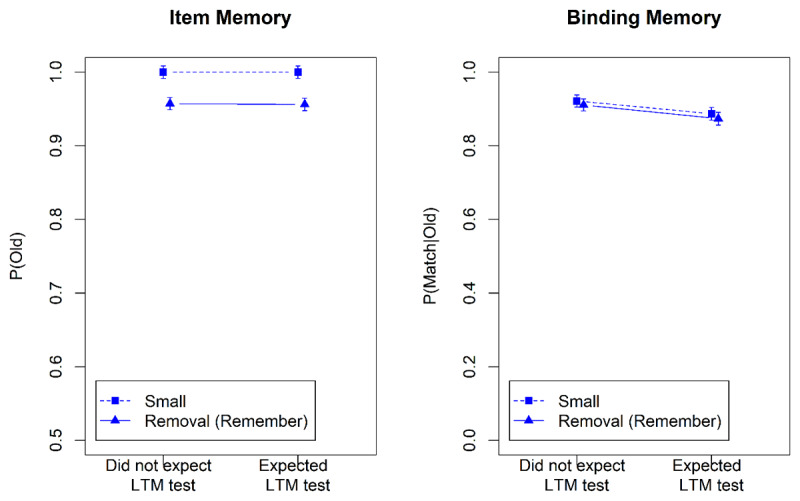
Accuracy in the Working-Memory Test of Experiment 5.

**Figure 8:**
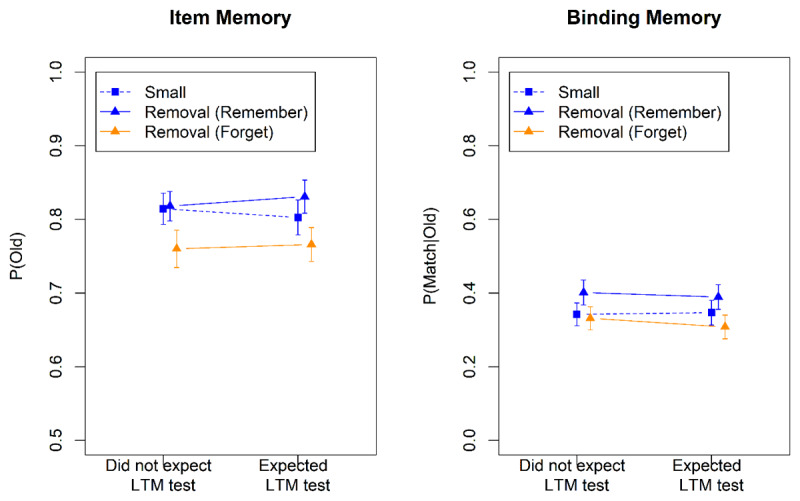
Accuracy in the Long-Term-Memory Test of Experiment 5.

Figures 7 and 8 show the accuracies in the WM and the LTM test, respectively. Table 5 summarizes the Bayes factors for the hypothesis tests. In the WM test, item memory in the removal condition was clearly worse than in the small set-size condition, indicating that removal of item memory was not perfect. By contrast, binding memory was indistinguishable between these conditions, showing virtually perfect removal of bindings. Whereas in Experiment 4 there was a tendency for binding memory to be removed less effectively when participants expected an LTM test, Experiment 5 did not replicate that.

**Table 5:** Bayes Factors for Working Memory and Long-Term Memory Performance in Experiment 5.


EFFECT	WORKING MEMORY		LONG-TERM MEMORY	
	
	ITEM MEMORY	BINDING MEMORY	ITEM MEMORY	BINDING MEMORY

Removal-Small	2605	0.25	0.18	0.13

Directed Forgetting			1299	26.1

Group	0.57	0.12	0.11	0.13

Removal-Small × Group	0.55	0.17	0.17	0.13

Directed Forgetting × Group			0.11	0.12


Whether or not participants expected an LTM test still had no discernible effect on their ability to remember the words in the long term. In particular, expecting the LTM test did nothing to mitigate the detrimental effect of removing information from WM on the ability to remember it in the LTM test. Although participants who expected an LTM test had 5 s between each word and the memory cue to establish that word in LTM, they did not (or were unable to) use that time effectively to improve LTM.

## Experiment 6

In the first five experiments we found that when participants remove information from WM, this leads to worse LTM performance for that information regardless of whether people expected the LTM test. In this final experiment we push the hypothesis that control of WM and LTM can be independent to its extreme. For each item we gave participants conflicting instructions – to remember items for the WM test but forget them for the LTM test (R_WM_F_LTM_ words) or vice versa (F_WM_R_LTM_ words). If people can control what they encode into their LTM separately from what they keep in WM, we should find that F_WM_R_LTM_ words are remembered better in the LTM test than R_WM_F_LTM_ words.

### Method

Methods, hypotheses, and the data analysis procedures were preregistered and can be found online (https://osf.io/9yrpg).

#### Participants

The same preregistered inclusion and exclusion criteria as in the preceding experiments were applied, and we only recruited participants that had not participated in the previous experiments. From the initial sample (*N* = 151), 43 participants had to be excluded (note, that 19 participants had to be excluded, because they had a mean error rate higher than 20%), resulting in a final sample size of *n* = 108 participants (*M*_age_ = 27.9, *SD*_age_ = 4.38, range 18–35 years; 45.4% female, 50.9% male, 3.7% other).

#### Material and Procedure

Stimuli and procedure were identical to Experiment 4 but we now instructed participants to forget words for the immediate memory tests when the frame turned orange but to remember only these words for the long-term memory (F_WM_R_LTM_ cues). At the same time, we instructed participants to remember the words followed by a blue frame only for the immediate but not the long-term memory test (R_WM_F_LTM_ cues). In the small set-size condition all words were followed by R_WM_F_LTM_ cues, whereas in the removal condition, between 3 and 5 of the 7 words were followed by R_WM_F_LTM_ cues and the remainder by F_WM_R_LTM_ cues.

### Results and Discussion

**Figure 9:**
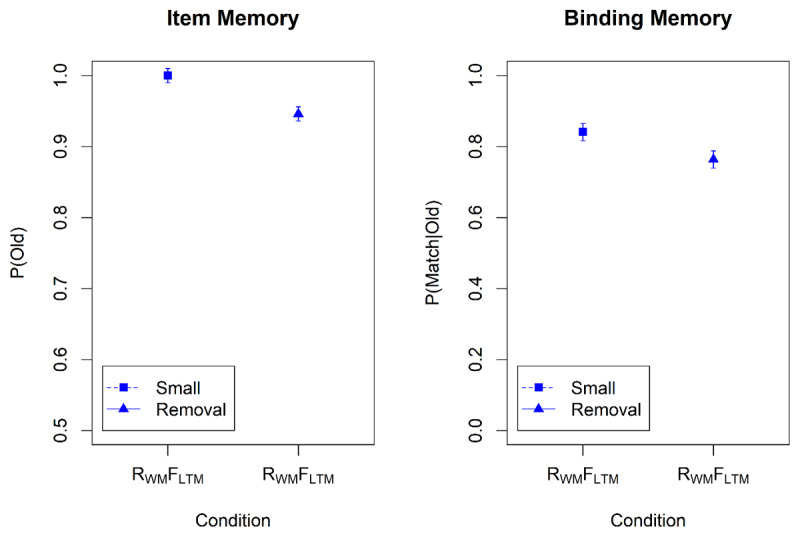
Accuracy in the Working-Memory Test of Experiment 6.

**Figure 10:**
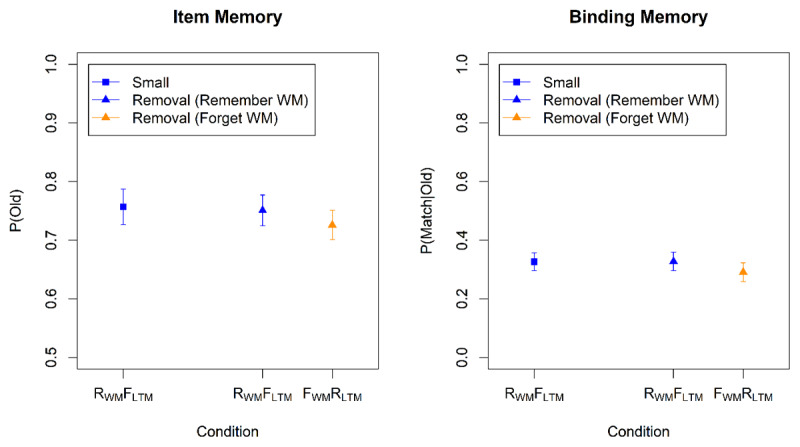
Accuracy in the Long-Term-Memory Test of Experiment 6.

The results for R_WM_F_LTM_ words in the WM test are shown in Figure 9. Both item memory (BF_10_ = 103) and binding memory (BF_10_ = 9.3) was worse in the removal condition than in the small set-size condition, indicating imperfect removal. The results from the LTM test are displayed in Figure 10. The words cued to be removed from WM but maintained in LTM (F_WM_R_LTM_ words) were remembered slightly worse than the words cued to be maintained in WM but forgotten in LTM (R_WM_F_LTM_ words). However, the statistical evidence for that difference remained ambiguous (BF_10_ = 0.50 and 0.32 for item and binding memory, respectively).

These results suggest that participants were unable to remove information selectively from WM while keeping it in LTM, or the other way around. When confronted with instructions to do so, they compromised: Words to be removed from WM were only partially removed. Still, doing so impaired LTM, but that impairment was less pronounced than in the preceding experiments.

## General Discussion

We asked whether people can selectively control what information to keep or not to keep in WM without affecting their long-term memory for that information. The answer is that they cannot. Across six experiments we successively increased our efforts motivating and enabling participants to remove information from WM while maintaining it in LTM, without success. When people remove an item from WM, they remember it less well in a subsequent test of LTM. The only way to mitigate that long-term effect is to hold back on removing the information from WM, as participants in Experiment 6 apparently did.

Our finding that people cannot control remembering in WM and in LTM independently might be related to a finding of Labaronne et al. (2024). They had participants remember word lists in a complex-span task. In each list one word was marked for a reward: Participants in one group were told that if they remembered that word in the WM test, they would receive a monetary reward; the other group was instead promised a reward for remembering that word in a delayed LTM test at the end of the experiment. When reward in the LTM test was promised, the rewarded word was recalled better than not-rewarded words in both the WM test and the LTM test. When reward was promised for the WM test, there was a statistically ambiguous tendency for it affecting also the LTM test. We can interpret the reward manipulation as a weaker version of a directed-forgetting instruction in which participants are encouraged to de-emphasize not-rewarded words but not completely forget them. One difference is that participants knew before list presentation which word will be rewarded and therefore could selectively attend to it already during encoding, whereas in the present experiments the R/F cues were presented only after encoding.

One possible explanation for the tight link between maintenance in WM and in LTM could be that after initial encoding the to-be-remembered items receive more attention than the to-be-forgotten items. The more we attend to some information the better it is established in LTM (Craik et al., 1996; Sasin & Fougnie, 2021). It is reasonable to assume that to-be-remembered words receive more attention than to-be-forgotten words. In the time between the memory cue and the next word, people are likely to keep attending to the preceding word if it was followed by an R cue but not if it was followed by an F cue. This should give to-be-remembered words an advantage in LTM. However, when we compensated for the shorter attention time of to-be-forgotten words through a longer SCI in Experiments 1 and 2, the to-be-forgotten words were still remembered worse than the to-be-remembered words. Regardless of the memory cue, the increase of the SCI, and with it the time during which words can be attended before the memory cue, did not measurably improve LTM.

This null effect is at odds with other findings showing that longer inter-item intervals in a WM test lead to better performance in a subsequent test of LTM (Labaronne et al., 2024; Loaiza & Lavilla, 2021; Loaiza & Souza, 2024; Souza & Oberauer, 2017). One possible reason is that the present SCI manipulation, extending the inter-item interval only by 1 s, may have been too weak to produce a measurable effect. A comparison of LTM performance between Experiments 4 and 5 supports that interpretation: The only difference between these experiments is the SCI, which we increased from 0 to 5 s. This substantial increase in time could be responsible for the much better LTM performance in Experiment 5 than in Experiment 4. If that is the case, then the beneficial effect of longer time for LTM must be independent of people’s intention to remember the words in the long term. This is because longer time lifted LTM performance regardless of whether participants expected the LTM test. We conclude that a longer dwell time of attention on an item likely improves LTM for it; however, the brief period of prolonged attention following an R cue appears too small to fully account for the directed-forgetting effect we observed in LTM.

To-be-remembered words could receive more attention than to-be-forgotten words not only in the interval immediately after their presentation, but also later during a WM trial. For instance, every time a word is followed by an F cue, a person could direct their attention to previously encoded to-be-remembered words that they hold in WM. This idea is closely related to the assumption of selective rehearsal of to-be-remembered words, which is one of the leading explanations of item-based directed forgetting in episodic LTM (Tan, Ensor, Hockley, Harrison, & Wilson, 2020); it is also related to the notion of attention-based refreshing in WM (Barrouillet, Bernardin, & Camos, 2004) and the assumption that refreshing improves LTM (Camos & Portrat, 2015). In the present experiments, there was at most a 1 s interval between the presentation of an F cue and the onset of the next word, leaving little time to attend to the previous words. Given that R and F cues occurred equally often, participants could gain, at most, 1 additional second of processing time for each to-be-remembered word. The null effect of the SCI manipulation in Experiments 1 and 2 makes it unlikely that just directing attention to to-be-remembered words for one additional second fully explains the directed-forgetting effect in LTM. However, directing attention back to previously encoded words could yield a greater memory benefit than simply continuing to attend to the just-presented word. This is because redirecting attention involves retrieving the word from WM, and perhaps also from LTM. Such retrieval practice may help explain why to-be-remembered words are maintained better in LTM than to-be-forgotten words.

One finding in the study of Labaronne et al. (2024) mentioned above is relevant for this question: They varied the time between presentation of successive words, and that manipulation did not interact with the effect of reward on WM and LTM performance. If people used free time to selectively attend to and retrieve the to-be-rewarded word, more time should lead to a larger LTM benefit of reward. The absence of that interaction speaks against the idea that attending back to an item previously encoded into WM enhances its representation in LTM. The effect of reward was more likely due to longer attention to the rewarded word during encoding than during maintenance.

Another study speaks more in favor of the assumption that attending to items in WM improves LTM for them: Labaronne, Caclin, and Plancher (2025) asked participants to remember lists of words or pseudowords for serial recall after a brief retention interval filled with parity judgments. Participants in one group were instructed to silently rehearse the items during the retention interval whereas another group was instructed to “think back” of the items without speaking them. Whereas the rehearsal instruction led to better immediate serial recall, the “think back” instruction led to better recall of words in a delayed free-recall test. On the assumption that “thinking of” involves more attention to the items than subvocal rehearsal, this result could reflect a beneficial effect of attending to items in WM on LTM. However, the two instructions might also differ in another relevant way: Whereas rehearsal engages phonological representations, thinking of a word probably engages its meaning, and processing the meaning of a word leads to better episodic LTM than processing its phonology (Craik & Lockhart, 1972).

A second approach to explaining the linked fate of memories in WM and LTM is based on assumptions about the mechanisms by which information is removed from WM. When a representation is removed from WM, it is somehow erased or altered to make it less accessible. For instance, in the SOB-CS model of WM (Oberauer et al., 2012), items are encoded into WM by binding them to their contexts. An item is removed from WM by unbinding it from its context. The context is any information that could serve as a retrieval cue for the item. In the present experiments, the items are the words, and the contexts are their locations on the screen, as well as a temporal context that distinguishes the current trial from previous trials, and the experimental setting from other events in the person’s life. Representations of items in episodic LTM also consist of bindings between the item and its context (Raaijmakers & Shiffrin, 1980; Sederberg, Howard, & Kahana, 2008). It is possible that WM and LTM use the same representations. If that is the case, then unbinding an item from its context to remove it from WM automatically unbinds that item from its context in LTM, rendering it less accessible in a test of LTM.

If the assumption of shared representations is not true, and instead WM and LTM form separate, largely parallel representations of the same events, then their fate could be coupled because the control signal that triggers the unbinding of an item from its context is not specific to the WM representation of that item but rather affects its parallel LTM representation as well. In that scenario, an F cue would set in motion a process that unbinds the representation of the just-encoded word from its context in WM, and because that process does not distinguish between WM and LTM, it also unbinds the word representation from its context in LTM.

To conclude, it is difficult and perhaps impossible to control the contents of WM and LTM independently. We cannot remove information from WM while making sure that we remember it later, and we cannot keep information in WM while keeping it out of LTM.

## Data Accessibility Statement

All experiments were preregistered prior to data collection. The data as well as experiment files and analysis codes are publicly available on OSF: https://osf.io/bfx3n.

## Footnotes

Or a mixture of the two, leading to a result pattern intermediate between the two scenarios.

For the effects tested in both analyses we report the more conservative BF estimate (i.e., the value closer to 1).
